# Increasing exercise participation during the COVID-19 pandemic: the buffering role of nostalgia

**DOI:** 10.3389/fpsyg.2023.1285204

**Published:** 2023-12-07

**Authors:** Heetae Cho, John Chee Keng Wang, Sunghoon Kim, Weisheng Chiu

**Affiliations:** ^1^Department of Sport Science, Sungkyunkwan University, Suwon, Republic of Korea; ^2^Department of Physical Education and Sports Science, Nanyang Technological University, Singapore, Singapore; ^3^Department of Physical Education, Yonsei University, Seoul, Republic of Korea; ^4^Lee Shau Kee School of Business and Administration, Hong Kong Metropolitan University, Hong Kong, Hong Kong SAR, China

**Keywords:** exercise, nostalgia, solastalgia, impulse buying, COVID-19

## Abstract

**Introduction:**

Due to the coronavirus disease 2019 (COVID-19) pandemic, people faced difficulties engaging in exercise activities as usual. As a result, there has been an increase in the demand for home exercises and online sales. However, there is little research on individuals’ buying and exercise behaviors during the pandemic. Thus, this study investigated how the perceived threat of COVID-19 influences exercise participants’ compensatory consumption and exercise intention through emotional responses, such as feelings of solastalgia and nostalgia.

**Methods:**

A total of 488 responses were collected from Generation Y, as individuals belonging to Generation Y are more prone to impulsive buying compared to other generations and, importantly, consider exercise a crucial component of their general well-being. Data were examined using a three-step method that involved the use of SPSS 26.0 and EQS 6.4 software.

**Results:**

Results showed that perception of COVID-19 positively influenced solastalgia and negatively affected nostalgia. Also, solastalgia had positive effects on nostalgia and online browsing, and nostalgia positively affected online browsing. Finally, this study found that online browsing positively influenced impulse buying and exercise intention, while impulse buying did not significantly affect exercise intention.

**Conclusion:**

This study contributes to identifying the crucial influence of emotions in decision-making and increasing the understanding of the connection between nostalgia and cognitive and emotional responses amid the COVID-19 pandemic.

## 1 Introduction

Since the global outbreak of the coronavirus disease 2019 (COVID-19) pandemic, many countries have rolled out different social distancing measures to slow down the spread of the disease, and people are encouraged to stay indoors and head out only for essential activities. When the World Health Organization (WHO) declared the COVID-19 outbreak a pandemic (World Health Organization, 2020), many countries tried to prevent the spread of the virus. For instance, to minimize the further spread of COVID-19, Singapore initiated lockdown measures in April 2020, mandating schools and workplaces to adopt home-based learning and work-from-home measures, while non-essential services were forced to close down temporarily (Ministry of Health Singapore, 2020). The lockdown measures have caused an economic slowdown and affected consumer spending, with retail sales showing a 40.5% plunge during the lockdown (Tan, 2020). Thereafter, Singapore embarked on a three-phase approach to resume activities safely, also known as post-lockdown (Singapore Government, 2020).

Majority of people spend most of their time working and learning from home during and post-lockdown, so their buying behaviors have changed. One includes shifting from in-person to online shopping (Chaudhary, 2020). This change in shopping patterns is likely to persist even after the country eases current restrictions as individuals adjust to a new normal in the post-COVID-19 world, which includes e-commerce (Guthrie et al., 2021). Despite the general fall in retail sales, individuals frequently purchase indoor exercise equipment, making it a popular product category for online shopping (Businesswre, 2020). This indicates that people have turned to indoor, do-it-yourself fitness activities, which resulted in a spike in the sales of indoor fitness equipment for their home workouts, leading to an over 70% increase in the sales of indoor fitness equipment during the lockdown period (Ng and Chen, 2020). Also, fitness equipment stores reported significant increases in sales and website traffic (Lee et al., 2020). This all points to the fact that many people have started to understand the importance of exercise and fitness amidst the COVID-19 outbreak.

In the academic field, researchers highlighted the importance of exercise in promoting individuals’ health and well-being, as it can alleviate stress and act as a coping mechanism (Lutz et al., 2010). However, during the COVID-19 pandemic, it was not easy for individuals to participate in outdoor exercise and fitness activities as before, diverting the demands to home exercises and online sales (Roggeveen and Sethuraman, 2020; Schnitzer et al., 2020). Despite the existing literature explaining individuals’ buying and exercise behaviors, little research has looked into sport product consumers’ impulse buying behavior and its relationship with exercise behavior during the COVID-19 pandemic. In addition, although researchers highlighted that emotions are significant antecedents of behavioral outcomes (Moors et al., 2013; Smith et al., 2014; Williams, 2014), the role of nostalgia (i.e., a sentimental longing for the past) and solastalgia (i.e., distress caused by environmental impacts) have not been investigated to understand individuals’ consumption and exercise behaviors in a pandemic situation. Therefore, this study examined the effects of the perceived COVID-19 threats on compensatory consumption behavior (i.e., impulse buying) and exercise intention through solastalgia and nostalgia based on appraisal theory (Lazarus, 1991). The findings of this study contribute to identifying the significant role of emotions in decision-making and help understand the relationship between consumption and exercise behaviors during the COVID-19 era.

## 2 Literature review and hypothesis development

### 2.1 Appraisal theory of emotion

The appraisal theory of emotion (Lazarus, 1991) asserts that the relationship between a person and the environment influences the onset of distinctive emotions. This specific set of emotions is activated through a thorough cognitive appraisal of a situation following an individual’s needs (Lazarus, 1991, 2001). In other words, emotions are not simply automatic responses to external events but result from a person’s appraisal or evaluation of a situation. For example, when an individual evaluates a situation as a threat, a specific emotion or combination of them is activated. These specific emotions do not necessarily come from a single cognitive content appraisal but could result from various appraisals. The appraisal theory of emotions also suggests that individuals intend to cope with adverse situations (Lazarus, 1991, 2001).

Applying the appraisal theory of emotion (Lazarus, 1991), it can be explained how the COVID-19 pandemic elicited a range of emotions in people, affecting behavioral responses. In the COVID-19 situation, people may appraise the pandemic as highly relevant to their health and well-being, leading to various emotions related to COVID-19. For example, people may feel fearful about the potential health risks of the virus and psychological distress about its negative environmental impacts (Zhang et al., 2020). In addition, when people appraise the current situation as highly stressful or threatening, they experience more intense negative emotions and have nostalgia due to various restrictions and uncertainty caused by COVID-19. Due to the evaluation of circumstances, individuals experience nostalgic feelings that make them recall memories of participating in sport activities (Cho, 2020).

**Figure 1:**
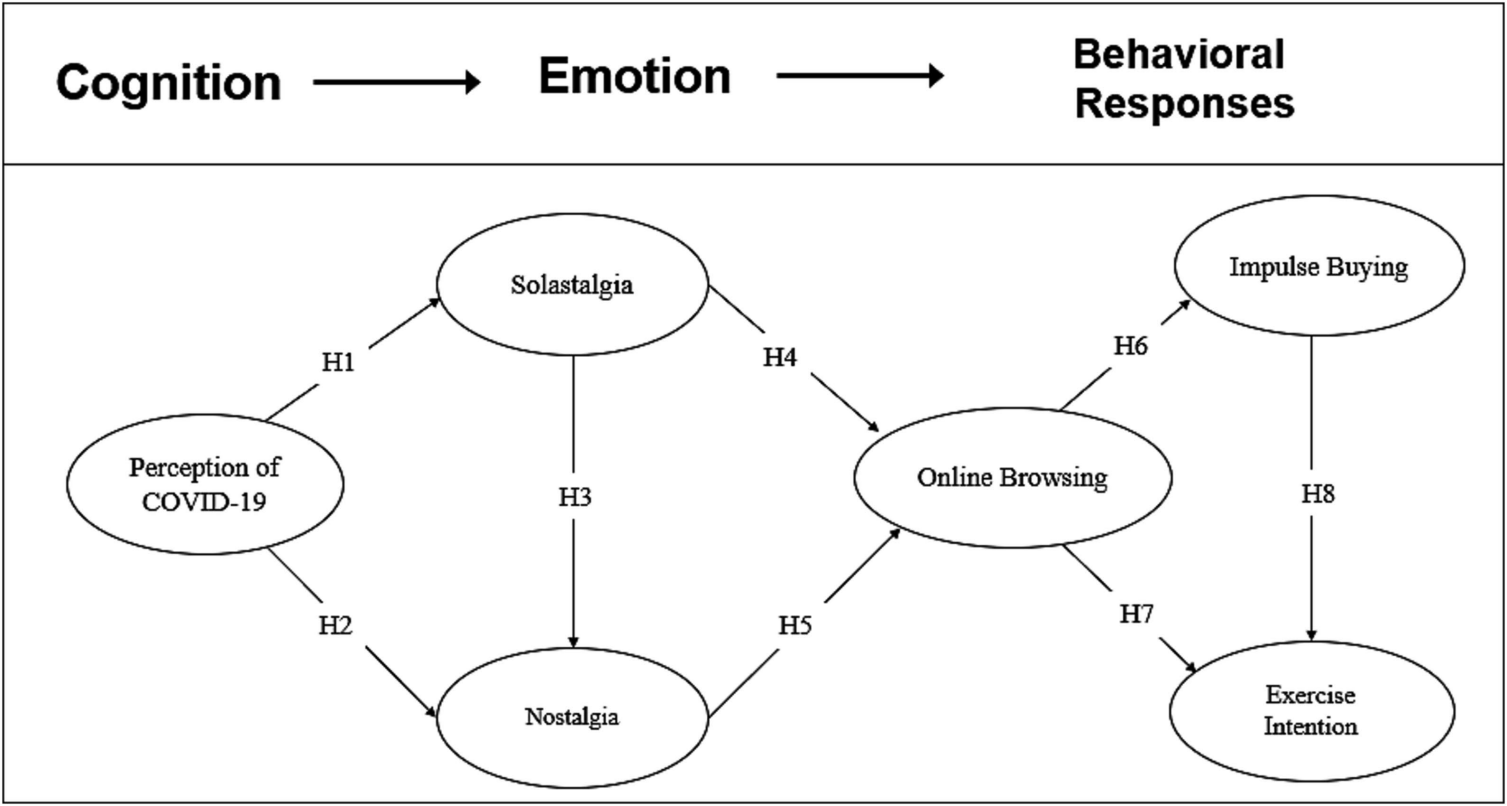
A hypothesized model.

This study considers solastalgia and nostalgia as specific emotions elicited by single or various cognitive appraisals (i.e., perception of COVID-19). In addition, Vess et al. (2012) noted that longing to relive past moments buffers the unhappiness felt in the present moment and encourages them to carry out a behavioral response according to the appraisal of their well-being. Therefore, this study examined individuals’ behavioral responses, such as online browsing, consumption, and exercise behaviors, which can be generated by emotions (i.e., nostalgia and solastalgia) (Lazarus and Smith, 1988), and suggest a hypothesized model (Figure 1).

### 2.2 Perceived risk of COVID-19, nostalgia, and solastalgia

The COVID-19 pandemic has led to significant changes in individuals’ daily lives, and they have become aware of the potential risks associated with the virus. Furthermore, perceived risk is essential in determining people’s psychological responses during the pandemic (Rather, 2021). Perceived risk of COVID-19 refers to an individual’s subjective assessment regarding the likelihood and severity of contracting the virus. When people perceive potential risks associated with COVID-19, they can experience various emotions, such as anxiety, fear, and stress (Levkovich and Shinan-Altman, 2020; Asmundson and Taylor, 2020a,b). Moreover, the COVID-19 pandemic has been linked to solastalgia. Albrecht (2005) coined the term solastalgia, defined as an individual’s psychological distress caused by environmental degradation. That is, solastalgia significantly changes the social and physical environments with which individuals are familiar. Due to COVID-19, people experience restrictions, such as lockdowns and social distancing measures, which could lead to isolation and disconnection from communities and environments, resulting in psychological distress (i.e., solastalgia) (Albrecht, 2005).

The COVID-19 pandemic has led to a resurgence of nostalgia in individuals, as people have the desire to return to the positive past and escape from the negative situation (Cho et al., 2014, 2019; Cho, 2023). In particular, during the pandemic, it was difficult to participate in sport as usual due to the restrictions, which generated nostalgic feelings regarding their sport experiences (Kaur et al., 2020; Shariat et al., 2020). In addition, previous studies noted that nostalgia buffers negative psychological responses (Juhl et al., 2010; Baldwin et al., 2018). For example, Stephan et al. (2014) examined the relationship between nostalgia, avoidance, and approach motivation and found that avoidance motivation generates nostalgic feelings, increasing approach motivation. Given the COVID-19 situation, nostalgia plays a role in buffering the feeling of distress caused by significant changes in the environment. Furthermore, Wang et al. (2023) found that nostalgia buffered cyberbullied feelings and promoted individuals’ psychological well-being. This indicates that nostalgia buffers against negative cognitive and emotional factors and leads to positive behavioral and psychological outcomes (Cho, 2023). Therefore, based on previous studies, this study suggests the following hypotheses:

H1: Perception of COVID-19 has a positive effect on solastalgia.

H2: Perception of COVID-19 has a positive effect on nostalgia.

H3: Solastalgia has a positive effect on nostalgia.

### 2.3 Online browsing

The COVID-19 pandemic led to significant changes in individuals’ behavior. In particular, during the pandemic, people are more likely to use internet sources to obtain information or content on their interests (Nguyen et al., 2020), which is motivated by emotional responses (Hausman, 2000; Huang, 2005; Yi and Jai, 2020). In other words, individuals turn to the Internet for information to fulfill their needs and adapt to a new way of life; emotions can affect browsing behavior (Park et al., 2012; Habib and Qayyum, 2018). Previous research found that with the pandemic causing widespread uncertainty, people experience negative emotions, such as fear and distress, increasing behavioral outcomes (Pakpour et al., 2020; Shechter et al., 2020; Chiu et al., 2022). That is, when people experience emotional distress, they may turn to the Internet as a coping mechanism or a way to distract themselves from their negative emotions. This indicates that emotional responses increase browsing behavior as a form of self-soothing or avoidance.

Moreover, Cho et al. (2021) noted that individuals’ nostalgia could play a buffering role during a pandemic and increase browsing behavior. Also, individuals can alleviate their negative emotions by searching for information on specific products of interest (Cho et al., 2021), indicating that nostalgia can motivate browsing behavior to connect with their positive past experiences. In sum, browsing behavior during COVID-19 can be influenced by a range of emotions, as people sought information, connection, entertainment, and ways to cope with the challenges of the pandemic. Therefore, based on previous studies, this study suggests the following hypotheses:

H4: Solastalgia has a positive effect on online browsing.

H5: Nostalgia has a positive effect on online browsing.

### 2.4 Impulse buying and exercise intention

Impulse buying refers to an individual’s consumption behavior of making unplanned purchases, which can be triggered by a sudden urge; it can occur in online and offline settings (Rook, 1987). Researchers noted that browsing behavior is crucial in impulse buying behaviors in online shopping environments (Zhang et al., 2018). During the COVID-19 pandemic, there were significant changes in online browsing behavior. People spend more time indoors and rely on the Internet for work and leisure (Nguyen et al., 2020; Cho et al., 2021). In particular, the convenience of online shopping makes it easier for them to purchase products on impulse without leaving their homes (Moe, 2003). When people engage in online browsing behavior, they are often motivated by the desire for enjoyable experiences and search for practical information or products to fulfill a specific need (Jarboe and McDaniel, 1987; Moe, 2003; Floh and Madlberger, 2013; Pöyry et al., 2013). Similarly, in the field of sport, Cho et al. (2021) found that online browsing for sport products is a significant factor in increasing impulse buying during the COVID-19 situation. That is, with concerns about the pandemic, individuals search for information related to the pandemic and become more focused on their health and wellness (Cho et al., 2021). This could lead to increased behavioral responses related to exercise, such as impulse buying of exercise products and intention to participate in the exercise. Therefore, this study proposes the following hypotheses:

H6: Online browsing positively affects the impulse buying of exercise products.

H7: Online browsing has a positive effect on exercise intention.

Last, Rusbult and her colleagues (Rusbult, 1980; Rusbult et al., 1998) noted that, based on the investment size, individuals’ level of commitment could increase, positively influencing intention to continue their behaviors related to their investment. During the pandemic, individuals are more likely to search for and browse exercise products due to a growing interest in health (Kaur et al., 2020). In this process, they invest their time, money, and emotional effort in exercise, and their lives may become linked to it (Rusbult et al., 1998; Cho et al., 2014, 2019). Given that exercise becomes a critical component of individuals’ lives, people can impulsively purchase exercise products (Stern, 1962; Zhang et al., 2018); due to the investment in exercise, they can develop exercise intention (Rusbult et al., 1998). Therefore, this study proposes the following hypothesis:

H8: Impulse buying of exercise products has a positive effect on exercise intention.

## 3 Methods

### 3.1 Research participants

This study collected data from individuals who belong to the Millennial population (Generation Y), as they are more susceptible to “impulse buying” than other people (Aruna and Santhi, 2016). In addition, Millennials showed the highest exercise participation rate compared to the other generations and considered exercise an integral part of their overall well-being (Alphagraphics, 2018; Statista, 2023). Thus, the prerequisite for research participation is that individuals belong to the Millennial population (Generation Y), born between 1982 and 1999 (Howe and Strauss, 2000). This study obtained Institutional Review Board (IRB) approval from the second author’s affiliated institution (IRB-2022-114) and collected data in August 2022. Specifically, the online survey was administered through Rakuten.com, and the platform provider offered approximately S$2 as compensation to each participant for their involvement in the survey. The information provided by the participants was anonymous, and this study had no intention of gathering identifiable details, such as name or IP address. A total of 488 participants were randomly selected to participate in a survey conducted in Singapore. Males comprised 48.8% (*n* = 238), and females comprised 51.2% (*n* = 250) of the respondents. The most highly reported age group was 33 to 38 (42.2%, *n* = 206), followed by 27 to 32 (36.5%, *n* = 178) and 21 to 26 (21.3%, *n* = 104). More than 70% had a bachelor’s degree (71.7%, *n* = 350), and a monthly household income of 50% was over S$7,000 (52.5%, *n* = 256). As for marital status, more than half of the respondents were single (never married) (53.5%, *n* = 261), followed by married (45.1%, *n* = 220), divorced (1%, *n* = 5), widowed (0.2%, *n* = 1) and separated (0.2%, *n* = 1). This study also asked whether they participated in any new leisure activities after the COVID-19 outbreak to identify leisure participation patterns and found that 54.1% (*n* = 264) of them participated in new leisure activities (e.g., cycling, wall climbing, badminton, hiking, and yoga).

### 3.2 Measures

The questionnaire consisted of seven sections: (a) the perception of COVID-19, (b) solastalgia, (c) nostalgia, (d) online browsing activities, (e) impulse buying behavior, (f) exercise intention, and (g) demographic information (e.g., gender, age, ethnicity, marital status, and household income). All items for each construct will be adopted and borrowed from the existing literature. Specifically, Lee et al. (2012) 4-item scale was used to measure the perception of COVID-19, and Eisenman et al. (2015) 6-item scale was modified and used to measure solastalgia. Next, Cho et al. (2019) leisure nostalgia scale was used to measure nostalgia. Before assessing nostalgia, this study used one item (i.e., Do you have any positive memories regarding your favorite fitness/exercise activity in the past?) that suggested positive recollections are necessary for experiencing nostalgia (*M* = 5.31, SD = 1.23). According to the result, 30 respondents answered that they do not have any positive memories regarding their favorite fitness/exercise activity; thus, their responses were not used for data analysis. The leisure nostalgia scale (Cho et al., 2019) consists of 33 items across five subfactors, including nostalgia as leisure experience, environment, socialization, personal identity, and group identity. To measure sport consumers’ online browsing activity, this study employed Beatty and Ferrell’s (1998) scale and modified it to suit fitness/exercise products. This study also used Harmancioglu et al. (2009) scale to measure sport consumers’ impulse buying behavior. Last, this study borrowed and modified three items used in Vlachopoulos and Michailidou’s (2006) study to measure exercise intention. A 7-point Likert-type scale was used to measure each construct.

### 3.3 Data analysis

In this study, data were examined using a three-step method that involved the use of SPSS 26.0 and EQS 6.4 software. The steps included: (a) screening of data, (b) conducting a confirmatory factor analysis (CFA), and (c) performing structural equation modeling (SEM). First, this study identified the univariate and multivariate outliers via significance testing with z-scores (−3.29 < z < 3.29) (Tabachnick and Fridell, 2013) and Mahalanobis distance (Penny, 1996). This study removed 13 univariate and five multivariate outliers according to the results. Thus, 440 responses were used for further data analyses. This study calculated the required sample size based on the population size, confidence level (95%), and margin of error (5%). This study found that the ideal sample size was 385, indicating that sufficient responses were collected for this study. Next, this study tested the normality assumption using Mardia’s (1985) multivariate kurtosis coefficients and found that the data did not hold it (Marida’s multivariate kurtosis coefficients = 46.62). As such, this study used the Satorra-Bentler scaled chi-square test (S-B $\chi^{2}$ ) and robust standard errors when interpreting the results of the SEM analysis (Bentler and Dijkstra, 1985; Satorra and Bentler, 1994). These techniques are considered more robust when non-normality is examined with a large sample (Bentler, 2005). Thereafter, this study conducted CFA with robust maximum likelihood estimation to evaluate the psychometric properties of the measures. This study employed root mean square error of approximation (RMSEA), standardized root mean squared residual (SRMR), non-normed fit index (NNFI), and comparative fit index (CFI) to assess the goodness-of-fit for the measurement model. As for the validity and reliability of the measurement model, Rho or composite reliability was reviewed, and AVE values were used for convergent validity (Fornell and Larcker, 1981). This study also compared the AVE values with the squared correlations among constructs to assess discriminant validity (Fornell and Larcker, 1981). Last, this study examined SEM analysis to assess the structural models for hypothesis testing (Anderson and Gerbing, 1988) by evaluating the data fit of the covariance–variance matrixes associated with the structural models and errors through multiple fit indices, including RMSEA, SRMR, NNFI, and CFI (Hu and Bentler, 1999; Kline, 2011).

## 4 Results

### 4.1 Measurement model

The data fit the initial measurement model: S-B *χ^2^*(*df*) = 590.43(215), CFI = 0.94, NNFI = 0.93, RMSEA = 0.06, and SRMR = 0.08. Next, this study evaluated the reliability of the measurement model based on the Rho coefficients and found that the Rho coefficients ranged from 0.80 for online browsing to 0.94 for solastalgia, indicating a satisfactory level of reliability. Next, the convergent and discriminant validity of the measurement model were evaluated. Specifically, the average variance extracted (AVE) values were utilized to assess convergent validity; this study compared the corresponding square root of the AVE values and corresponding correlations to test discriminant validity. The results showed that all six constructs had AVE values above 0.50, suggesting satisfactory levels of convergent validity (Fornell and Larcker, 1981) (Table 1). In addition, this study found that the correlations between the constructs were lower than the respective square roots of the AVEs, indicating acceptable levels of discriminant validity (Table 2) (Fornell and Larcker, 1981).

**Table 1:** Factor loading (λ), Rho, and AVE values for the improved model.

Factors and items	*λ*	Rho	AVE
*Perceived risk of COVID-19* (*M* = 4.10, *SD* = 1.46)		0.89	0.68
It is dangerous to go out because of COVID-19	0.82		
COVID-19 is a very frightening disease	0.88		
Compared to SARS and H1N1, COVID-19 is more dangerous	0.74		
I am afraid of COVID-19	0.85		
*Solastalgia* (*M* = 4.37, *SD* = 1.37)		0.94	0.71
Seeing my exercise participation affected by COVID-19 has been stressful	0.79		
I have gone to my favorite exercise place affected by COVID-19 less than I did before the COVID-19 situation	0.71		
I feel like I have been grieving the loss of my exercise participation affected by COVID-19	0.88		
I feel sad when I look at my exercise participation damaged by COVID-19	0.89		
I feel that aspects of exercise that I value were lost after the COVID-19 outbreak	0.90		
Unique aspects of my exercise were lost after the COVID-19 outbreak	0.87		
*Nostalgia* (*M* = 5.00, *SD* = 0.94)		0.93	0.72
Sport team	0.82		
Environment	0.80		
Socialization	0.82		
Personal identity	0.91		
Group identity	0.89		
*Online browsing* (*M* = 4.58, *SD* = 1.26)		0.80	0.58
The amount of time I spent just searching for fitness/exercise products on e-commerce websites was fairly high	0.84		
When I browse e-commerce websites for fitness/exercise products, I would say that I was primarily “just looking around	0.53		
I devoted most of my attention to fitness/exercise products I planned to buy on e-commerce websites	0.86		
*Impulse buying* (*M* = 4.18, *SD* = 1.59)		0.92	0.85
I ended up spending more money for fitness/exercise products than I originally set out to spend	0.92		
I bought more fitness/exercise products than I had planned to buy	0.92		
*Exercise intention* (*M* = 5.42, SD = 1.05)		0.91	0.79
I intend to continue participating in my favorite exercise	0.89		
I will try to continue participating in my favorite exercise	0.89		
I am determined to continue participating in my favorite exercise	0.88		

AVE, average variance extracted.

**Table 2:** Values of correlation and squared root of AVE among all factors.

	(1)	(2)	(3)	(4)	(5)	(6)
(1) Perceived risk of COVID-19	0.82^1^					
(2) Solastalgia	0.36^*^	0.84^1^				
(3) Nostalgia	0.08^*^	0.52^*^	0.85^1^			
(4) Online browsing	0.19^*^	0.47^*^	0.60^*^	0.76^1^		
(5) Impulse buying	0.20^*^	0.52^*^	0.53^*^	0.74^*^	0.92^1^	
(6) Exercise intention	−0.04^*^	0.28^*^	0.64^*^	0.34^*^	0.28^*^	0.89^1^

^1^Squared root of AVE, ^*^p < 0.05.

### 4.2 Structural model

The data also fit the structural model well: S-B *χ^2^*(*df*) = 706.77(222), CFI = 0.93, NNFI = 0.92, RMSEA = 0.07, and SRMR = 0.08. This study conducted a structural equation modeling (SEM) analysis to test the proposed hypotheses. The results showed that perception of COVID-19 positively influenced solastalgia (H1: *β* = 0.36, SE = 0.05, *p* < 0.001) and negatively affected nostalgia (H2: *β* = −0.12, SE = 0.03, *p* < 0.01). This indicates that the perception of COVID-19 caused distress and made people less likely to feel nostalgia. Next, solatalgia had positive effects on nostalgia (H3: *β* = 0.57, SE = 0.04, *p* < 0.001) and online browsing (H4: *β* = 0.25, SE = 0.06, *p* < 0.001). These results indicated that when people experience emotional distress caused by negative environmental change, they are more likely to long for the past and browse online websites to search for sport-related products. This study found that nostalgia positively affected online browsing (H5: *β* = 0.54, SE = 0.11, *p* < 0.001), indicating that a predominantly positive emotion makes people browse e-commerce websites for fitness/exercise products. The results also showed that online browsing positively influenced impulse buying (H6: *β* = 0.77, SE = 0.06, *p* < 0.001) and exercise intention (H7: *β* = 0.58, SE = 0.09, *p* < 0.001), while impulse buying did not have a significant effect on exercise intention (H8: *β* = −0.14, SE = 0.07, *p* > 0.05). That is, online browsing increases people’s impulse buying behavior and exercise intention. However, individuals impulsively purchasing exercise products did not intend to participate in the exercise (Table 3).

**Table 3:** Results of regression and mediation analysis.

Path	*β*	SE	*z*-value
H1: Perception of COVID-19 → Solastalgia	0.36	0.05	6.36^***^
H2: Perception of COVID-19 → Nostalgia	−0.12	0.03	−2.78^**^
H3: Solastalgia → Nostalgia	0.57	0.04	8.74^***^
H4: Solastalgia → Online browsing	0.25	0.06	4.51^***^
H5: Nostalgia → Online browsing	0.54	0.11	8.27^***^
H6: Online browsing → Impulse buying	0.77	0.06	14.92^***^
H7: Online browsing → Exercise intention	0.58	0.09	4.64^***^
H8: Impulse buying → Exercise intention	−0.14	0.07	−1.27

^**^p < 0.01; ^***^p < 0.001.

## 5 Discussion

In marketing research, emotions play a pivotal role in steering consumer behavior. Previous research suggested that delving into the nuanced facets of emotions is crucial, considering that each emotion has a distinct set of appraisals (Lazarus, 1991, 2001). This study marks the first to look into solastalgia and nostalgia as two distinct emotions influenced by the perception of the threat of COVID-19, which leads to impulse buying behavior and exercise intention through online browsing. This study proposed a hypothesized model encompassing eight hypotheses.

### 5.1 Theoretical implications

The results support the positive relationships between the perception of COVID-19 (H1) and solastalgia and between solastalgia and nostalgia (H3). As opposed to nostalgia, solastalgia is a new concept developed to represent distress produced by environmental change (Albrecht, 2005). As people are experiencing helplessness or a lack of control over the COVID-19 situation while they are at home, it is natural that people report higher distress when they perceive more significant threats of the pandemic. While nostalgia and solastalgia are distress triggered by a compromised sense of place, it is not surprising that both are positively associated.

The result showed a negative effect of the perception of COVID-19 on nostalgia, contrary to H2, although it was predicted that the relationship should be positive. Previous studies have shown that the COVID-19 pandemic has increased nostalgia as people desire to return to the positive past and escape from the negative situation (Cho et al., 2014; Cho, 2023). However, this study showed that when people perceive their current situations as out of their control and cannot achieve their desired outcomes, this may not increase the feeling of nostalgia (Cho et al., 2019; Cho, 2020). Another reason could be the government’s long movement restrictions during the data collection period. It was more than 2 years since the start of the pandemic, and there were various tightening and changes in keeping social distancing.

The findings of the current study support that feelings of solastalgia (H4) and nostalgia (H5) have positive effects on online browsing. These findings are consistent with previous studies in that emotional responses increase browsing behaviors as a form of coping or self-comfort (Cho et al., 2021). This study also shows that nostalgia has a more substantial positive effect on online browsing than solastalgia, indicating that nostalgia could be a more potent motivating factor in browsing behavior to connect with positive past experiences. In addition, due to the restrictions imposed by the pandemic, such as working from home or online lessons, people tend to spend much more time with their computers accessing information, leisure, socializing, and browsing online. It was also hypothesized that online browsing positively affects the impulse buying of exercise products (H6) and exercise intention (H7). The findings showed that both hypotheses were supported. It is known that browsing behaviors lead to impulse buying of sport products (e.g., Cho et al., 2021), particularly during the pandemic when people are more concerned with their physical and mental health. This, at the same time, could increase their intentions to exercise. Després (2021) suggests that obesity, sedentary behaviors, and a lack of physical activity should be targeted by health authorities to reduce the risk of severe COVID-19 outcomes. Many health ministries, including the Ministry of Health in Singapore, have acknowledged that obesity, diabetes mellitus, and other cardiovascular diseases could increase the risk of severe COVID-19 outcomes. Therefore, during the COVID-19 pandemic, individuals are more likely to browse exercise products, which leads to an increase in impulse buying of exercise products and a greater intention to exercise.

The final hypothesis predicted that impulse buying of exercise products positively affects exercise intention (H8). However, the results showed no significant effect of impulse buying of sport products on exercise intention. Typically, when people invest time and money in purchasing sport products, they should develop a stronger intention to exercise (Rusbult et al., 1998). Three explanations are possible. First, according to the transtheoretical model of change (Prochaska and DiClemente, 1983), buying sport products could represent the preparation stage; it is still one step away from the action stage, and sometimes this may not happen if the previous steps have not been given enough thought and time. For example, impulse buying can happen when there is a discount or because the product captured their interest and they have not considered exercising seriously. Second, during the COVID-19 pandemic, there were delays in shipping and freight services; the UNCTAD (2020) estimated a decline of 4.1% in maritime trade due to the unprecedented disruption caused by COVID-19. Finally, the data was collected from March to April 2022, when Singapore took a decisive step to ease COVID-19 restrictions toward normalcy. At this time, many people are allowed to go back to work, which may lower their intention to exercise.

The present study applies the appraisal theory of emotion (Lazarus, 1991) to explain how perceptions of the COVID-19 pandemic elicit different emotions and behavioral responses. A hypothesized model was proposed and tested. This evidence suggests that the perception of COVID-19 positively predicted solastalgia but negatively predicted nostalgia. Both solastalgia and nostalgia positively predicted online browsing and subsequent impulse buying of sport products and exercise intention. This shows that the appraisal theory of emotions is valid for explaining how the pandemic triggers different emotions and behavioral responses among individuals. The findings also suggest that it may harm nostalgia when people are in a negative situation for a prolonged period (such as in the current pandemic). This is the first study to concurrently examine nostalgia and solastalgia as two different emotions elicited by the distress caused by environmental impact. It was found that the two emotions are positively correlated; this implies that when people experience negative emotions (such as solastalgia), they may have nostalgic feelings (such as good memories they had in sport).

### 5.2 Practical implications

Based on the findings of the current study, this study suggests several practical implications. First, a life-threatening event, such as the COVID-19 pandemic, may elicit different emotional responses (i.e., nostalgia and solastalgia), leading to various individuals’ behavioral responses (online browsing, impulse buying, and exercise intention). Therefore, fitness professionals and health organizations may want to evaluate people’s emotions influenced by the COVID-19 pandemic when promoting physical activities. For example, fitness professionals can provide online fitness offerings, such as live-streaming classes or recorded fitness videos, for consumers to exercise at home. Moreover, health organizations and fitness professionals should design exercise programs that cater to different exercise intentions (maintenance, weight loss/gain, or stress relief), which different emotional responses to the pandemic can influence.

Creating engaging exercise content and promoting it through social media platforms can attract potential clients who may be hesitant to participate in conventional exercise programs due to COVID-19. Furthermore, this study suggests that online platforms can significantly motivate individuals to engage in physical activity during pandemics by providing constant support and feedback. For example, using online platforms to promote exercise, set goals, track progress, and encourage social support can enhance individuals’ intention to exercise. Moreover, fitness professionals and health organizations should pay attention to online browsing behavior due to its impact on exercise participation.

Finally, from the perspective of marking, marketers of fitness products and service providers should understand the impact of COVID-19 on consumers’ emotions and behaviors, particularly in impulse buying and exercise participation. Therefore, marketers should design strategies to promote fitness products and services that cater to different emotional responses to the pandemic. These can be achieved by developing targeted advertisements and social media posts that appeal to specific emotional responses, such as nostalgia or solastalgia. In addition, the study suggests that marketers should highlight the benefits of exercise and physical activity in managing stress, anxiety, and other negative emotional responses to pandemics.

### 5.3 Limitations and suggestions for future research

There are a few limitations in this study that need to be acknowledged. First, this is a cross-sectional study, and therefore future studies can consider using experimental design or longitudinal design to determine the causal relationships among the key variables. Second, the study only selected Generation Y (born between 1982 and 1999), so the findings may not be generalized to other populations. Future research could include participants from other generations to increase the generalizability of the findings. In addition, given the rapid development of technology, future research could explore how technological advancements have affected the behaviors and attitudes of different generations. Last, this study was conducted during prolonged restrictions by the government due to the COVID-19 pandemic; it should be noted that during routine situations, the processes and effects may differ. With the widespread availability of vaccines and other public health measures, future research could investigate their impact on individuals and society. Further, researchers can examine the impact of prolonged restrictions on mental health, social relationships, and economic outcomes over a more extended period.

## Data availability statement

The raw data supporting the conclusions of this article will be made available by the authors, without undue reservation.

## Ethics statement

The studies involving humans were approved by Institutional Review Board of Nanyang Technological University (IRB-2022-114). The studies were conducted in accordance with the local legislation and institutional requirements. Written informed consent for participation in this study was provided by the participants’ legal guardians/next of kin.

## Author contributions

HC: Conceptualization, Funding acquisition, Investigation, Project administration, Writing – original draft, Writing – review & editing. JW: Writing – original draft. SK: Conceptualization, Methodology, Validation, Writing – original draft, Writing – review & editing. WC: Conceptualization, Formal analysis, Methodology, Writing – original draft, Writing – review & editing.
